# Tracer Diffusivity in Amphiphilic Polymer Model Co-Networks

**DOI:** 10.1021/acs.macromol.5c02458

**Published:** 2026-01-16

**Authors:** Sebastian Seitel, Lynn K. R. J. Zank, Stephanie Ihmann, Frank Böhme, Michael Lang, Bradley D. Olsen, Sebastian Seiffert

**Affiliations:** † Department of Chemistry, Johannes Gutenberg University Mainz, D-55128 Mainz, Germany; ‡ Department of Chemical Engineering, 2167 Massachusetts Institute of Technology, Cambridge, Massachusetts 02139, United States; § 28408 Leibniz-Institut für Polymerforschung, 01069 Dresden, Germany; ∥ Organic Chemistry of Polymers, Technical University Dresden, 01062 Dresden, Germany

## Abstract

Amphiphilic polymer conetworks (APCNs) are highly interesting material for membranes, drug delivery, or tissue engineering since their heterogeneous structure and interactions allow for the control of the diffusion of molecules differing by architecture, size, and interactions. We investigate the diffusion of hydrophilic and hydrophobic star polymers in model APCNs formed by heterocomplementary end-linking of tetra-poly­(ethylene glycol) (t-PEG) and tetra-poly­(*ε*-caprolactone) (t-PCL). Using Fluorescence Recovery After Photobleaching (FRAP) and Forced Rayleigh Scattering (FRS), we gain complementary insights into star polymer transport across different length and time scales. We compare the diffusion of hydrophilic t-PEG and hydrophobic t-PCL of various molecular weights across a wide range of APCN polymer volume fractions, swollen in a cosolvent (toluene) and a selective solvent (water). FRS reveals Fickian diffusion for all tracers in APCNs swollen in toluene. In the unentangled regime, the diffusivity of the tracer follows approximately the expected Rouse scaling for semidilute solutions. Corrections arise for increasing polymer content due to enforcing contacts with the other type of polymer in the APCN. At larger concentrations, the PEG tracers develop a diffusion behavior, as expected for entangled star polymers. Since the transition occurs below the expected entanglement concentration, an additional impact of the strangulation regime is likely. Partial swelling in a selective solvent leads to an enhanced diffusion behavior as compared to a homogeneously swollen network at the same polymer volume fraction; however, the concentration dependence of diffusion agrees best with the strangulation regime, despite an overall enhanced diffusion. At swelling equilibrium in the selective solvent water, the equilibrium degree of swelling, the network morphology, and the diffusion behavior become independent of the preparation conditions. These findings provide insights into the diffusion mechanism of star polymers within APCNs and contribute to the development of polymer-based drug delivery systems for biomedical applications.

## Graphical abstract

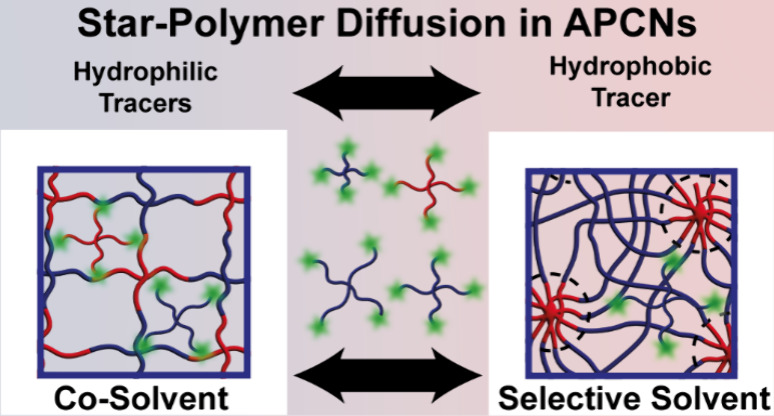

## Introduction

Amphiphilic polymer conetworks (APCNs) comprise hydrophilic and hydrophobic components, enabling environmentally sensitive transport and release of hydrophilic and hydrophobic solutes. This ability renders them exciting for use as membrane materials for drug or nutrient delivery,^1,2^ tissue engineering,^3^ artificial pancreases,^4^ and soft contact lenses as the most common application of APCNs so far.^5^ Potential further applications include matrixes for gel polymer electrolytes in batteries,^6^ phase-transfer enzymatic catalysis,^7^ antibiofouling surfaces,^8,9^ and pH sensing.^10^ APCNs are particularly relevant in biological and biomedical contexts due to their biocompatibility, tunable biodegradability, nontoxicity, and responsiveness to environmental stimuli.^11^ In addition to enabling the transport of both hydrophilic and hydrophobic substances, APCNs offer the advantage of reduced swelling under physiological conditions compared to conventional hydrogels, which helps maintain their mechanical properties.^12^ Among others, APCNs consisting in part of poly­(ethylene glycol) (PEG) or poly­(*ε*-caprolactone) (PCL) have often been used in studies focusing on biomedical applications.^12–15^ One common feature that most of these applications rely on is the efficient and controlled diffusion of small molecules, proteins, or polymers within the amphiphilic network system.

To date, the diffusion and release of small molecules, hydrophilic and hydrophobic drugs, peptides, and proteins in amphiphilic polymer conetworks have been widely studied.^16,17^ More detailed investigations have shown that transport in APCNs is strongly influenced by the network architecture and amphiphilic interactions. For example, Dech et al. have shown that interactions with the hydrophilic–hydrophobic interfaces of APCNs can mediate protein diffusion.^18^ Similarly, Löser et al. studied the diffusion of hydrophilic dextrans and hydrophobic linear polystyrenes in model PEG–PCL APCNs, identifying length scales such as the hydrodynamic screening length and correlation length in governing polymer transport. Notably, in selective solvents such as water, collapsed hydrophobic domains were shown to act as localized obstacles without significantly impeding diffusion.^19^ Beyond these mechanistic insights, the amphiphilic character of the networks further enables the codelivery of hydrophilic and hydrophobic substances. For example, Bera et al. synthesized a pH-responsive APCN, demonstrating the ability to entrap and release both hydrophilic and hydrophobic drugs,^20^ while PEG–PCL APCNs investigated by Yuan et al. have shown dual-release of choline theophyllinate and 5-fluorouracil, where network composition controls release rate.^21^ In general, studies on the release of small molecules from APCNs have demonstrated delayed release profiles and even zero-order kinetics for various drugs,^22,23^ providing strategies to overcome drug resistance and improve therapeutic efficacy in treatments, such as cancer therapy.^24^ Despite these advances highlighting the therapeutic potential and versatility of APCNs, the controlled release of polymer-conjugated drugs from covalently cross-linked APCNs remains an area that has not yet been extensively explored. This represents a significant opportunity for both applied and fundamental research. Hydrophilic PEG, widely used in PEGylation, can extend drug half-life, reduce immunogenicity, and improve targeting through stealth effects,^25–32^ with molecular weight and branching playing critical roles.^33^ In contrast, hydrophobic polymers such as PCL offer biodegradability, biocompatibility, and ease of functionalization.^34^ A mechanistic understanding of polymer mobility within APCNs is essential to enable predictive control over the transport properties in these systems. This need is particularly relevant given the growing role of macromolecular and polymer-conjugated therapeutics in modern medicine.^35–42^ In contrast to small molecules, such macromolecular species exhibit more complex transport characteristics in polymer networks, often governed by structural features of the network.^43,44^ Yet, how structural parameters of the network and the properties of the diffusing polymers govern transport in APCNs remains insufficiently understood. In particular, there is a lack of systematic studies examining how hydrophilic versus hydrophobic polymer tracers diffuse in amphiphilic networks and how the network composition and solvent quality modulate their mobility. Understanding these effects is crucial for designing networks that enable the controlled, selective transport of polymeric therapeutics in APCNs. Well-defined star polymer tracers are ideally suited to address this gap, since their branched architecture is biologically relevant,^33^ and their diffusion is highly sensitive to network structure and solvent interactions, allowing direct probing of selective transport mechanisms. Furthermore, since star polymers have shown to experience stronger retardation with increasing polymer volume fraction of a surrounding polymer matrix than linear polymers,^45^ they provide access to a broader range of diffusion coefficients and thus allow for more mechanistic insights, although this also makes quantitative interpretation slightly more complex. Bridging this knowledge gap is essential not only to rationally design APCNs with tailored transport properties for emerging therapeutic strategies but also to deepen the fundamental understanding of the transport of macromolecular components in structured soft materials.

The present work investigates the diffusion of hydrophilic tetra-poly­(ethylene glycol) (t-PEG) and hydrophobic tetra-poly­(*ε*-caprolactone) (t-PCL) inside a model PEG–PCL APCN in the semidilute regime to provide a basis for understanding structure–transport relationships. Recently, Bunk et al.^46^ extended the heterocomplementary tetra-PEG (t-PEG) coupling approach popularized by Sakai et al.^47^ to create such a model APCN through a heterocomplementary coupling reaction between amine-functionalized t-PEG and 2-(4-nitrophenyl) benzoxazinone-terminated t-PCL, yielding a well-defined model network structure (Figure 1). Using this approach, we employ a series of tracers, including t-PEG with molecular weights of 5, 10, and 20 kDa and t-PCL with 10 kDa; notably, PEG in the range of 400 to 50000 Da is commonly employed in biomedical systems.^33^ Diffusion is studied across a broad range of polymer volume fractions to identify transitions in diffusion mechanisms and compare them to existing theoretical models. Additionally, we explore how a change of the solvent quality from cosolvent toluene to the selective solvent water modulates the tracer polymer diffusion. The aim is to establish structure–transport relationships in APCNs and enable the predictive design of polymer-based functional soft materials.

**Figure 1:**
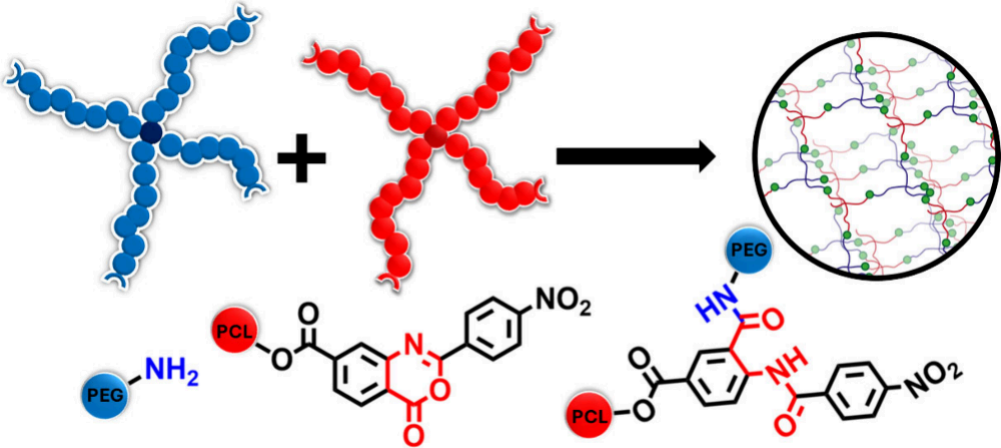
Schematic presentation of heterocomplemetary coupling reaction of amino-terminated t-PEG and 2-(4-nitrophenyl) benzoxazinone-terminated t-PCL.

To carry out our studies, we use Fluorescence Recovery After Photobleaching (FRAP) and Forced Rayleigh Scattering (FRS), providing complementary insights into the transport of polymeric solutes on different lengths and time scales. FRAP has become a standard technique for characterizing the diffusivity of fluorescently labeled solutes and tracers within a swollen polymer network matrix^48–52^ by tracking the fluorescence recovery after creating a locally bleached region. This method is well-suited for determining diffusion, interaction, or binding properties on the micrometer scale in biological and material sciences.^53^ In contrast, FRS relies on the interference pattern of laser-induced periodic photobleaching, allowing for a precise determination of the diffusion coefficient over a broad range of length scales and enabling the study of transport mechanisms. Furthermore, it allows the detection of anomalous diffusion such as sub- or superdiffusion. Together, these techniques elucidate how the polymer architecture, mesh size, and possible interactions influence the tracer mobility, giving a profound understanding of transport in complex soft materials. Since FRAP and FRS require dye-labeled tracers, we use t-PEG and t-PCL labeled with the fluorescent dye nitrobenzofurazan (NBD), which has proven suitable for both methods.^54–56^ With that, we make use of the ability to observe a small fraction of tracer polymers with just moderate degrees of chemical modification by the labeling but otherwise the same constitution as the surrounding matrixes.

## Materials and Methods

### Materials

t-PEG–OH is purchased from Biochempeg Scientific. *p*-Nitrophenylchloroformate and triethylamine are purchased from Sigma-Aldrich. (*S*)-(+)-4-(3-Amino-pyrrolidino)-7-benzofurazan is purchased from TCI. Toluene (≥99.5%) is purchased from Fisher Chemical. Dichloromethane (99.8%), extra dry over molecular sieves, is purchased from Thermo Fisher Scientific. Pyridine (99.8%), extra dry over molecular sieves, is purchased from Acros Organics. Milli-Q water is produced in an in-house Milli-Q system from Merck. All commercially available chemicals are used without further purification.

Synthesis of amino-terminated tetra-arm poly­(ethylene glycol) and 2-(4-nitrophenyl)-benzoxazinone-terminated tetra-arm poly­(*ε*-caprolactone) is performed as published elsewhere.^46^ Briefly, starting from commercially available 10 kDa t-PEG–OH, the terminal hydroxy groups are first reacted with mesyl chloride to yield a better leaving group for the following nucleophilic substitution with ammonia to give amino-terminated t-PEG. Ten kDa t-PCL–OH (10 kDa) is synthesized starting from pentaerythritol using *ε*-caprolactone, which is polymerized by ring-opening polymerization using Sn­(oct)_2_ as a catalyst. In the second step, t-PCL–OH is then converted to 2-(4-nitrophenyl)-benzoxazinone-terminated t-PCL by reaction with 2-(4-nitrophenyl)-4-oxo-4*H*-benzo­[*d*]­[1,3]­oxazine-7-carboxylic acid chloride, which is synthesized as described earlier.^57^ The obtained t-PCL–OH from the first reaction step is also used for NBD-functionalization.

### Synthesis of NBD-Labeled Tracers

To study tracer diffusion by FRS and FRAP, both t-PEG and t-PCL tracers are functionalized with a fluorescent dye on each arm by adapting a synthesis as detailed elsewhere.^55^ Briefly, t-PEG–OH and t-PCL–OH are activated by reaction with *p*-nitrophenylchloroformate. Further reaction with (*S*)-(+)-4-(3-amino-pyrrolidino)-7-benzofurazan (NBD) gives the final product (Scheme 1). Excess dye is removed by dialysis against Milli-Q water. An exemplary ^1^H NMR spectrum of 5 kDa t-PEG-NBD is shown in Figure S1, confirming successful synthesis together with the intense orange color of all obtained final products.

**Scheme 1:**
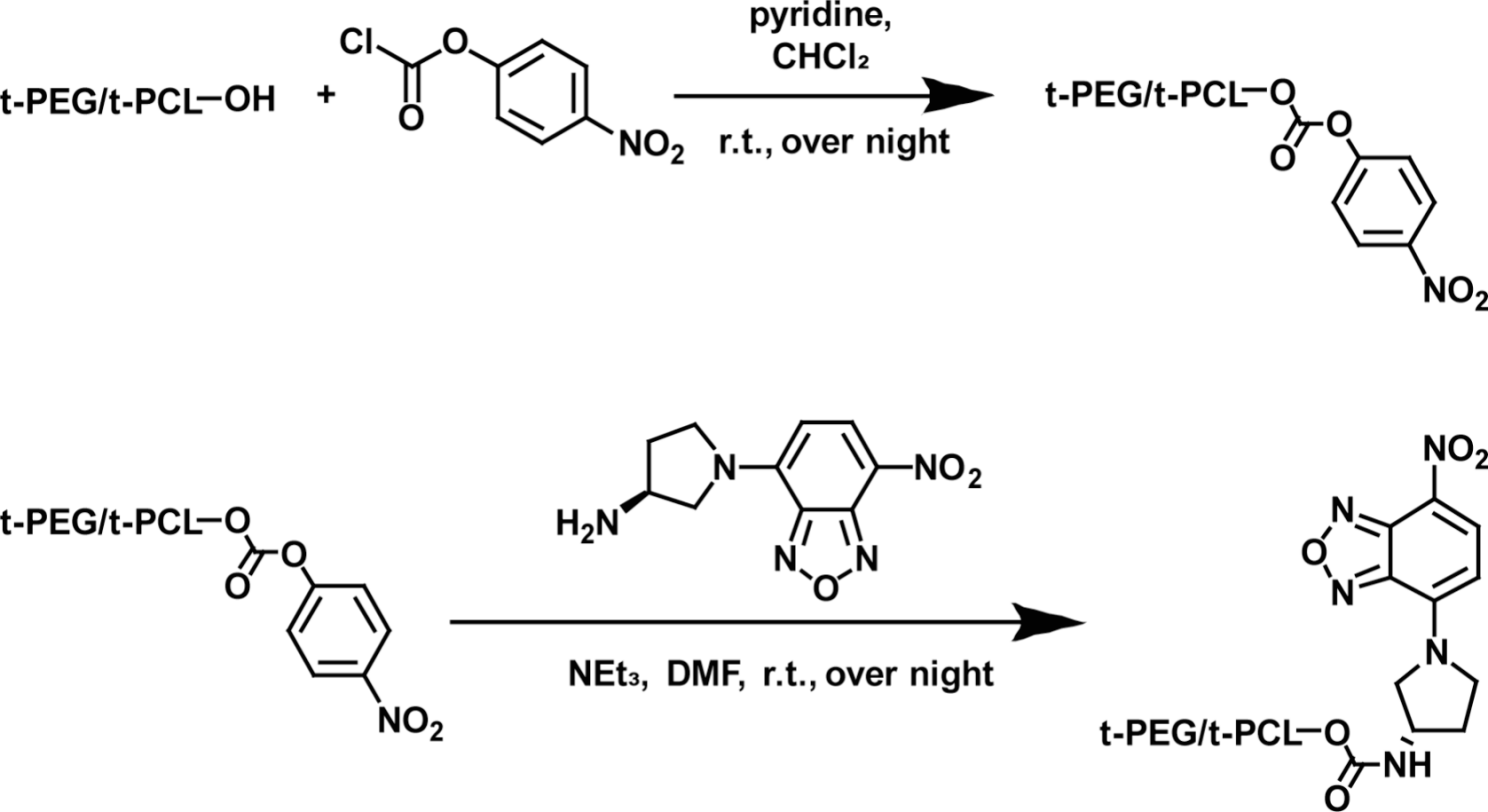
Synthesis of NBD-labeled t-PEG–OH and t-PCL–OH

### Formation of APCNs

For homogeneous APCN preparation, equimolar solutions of t-PEG-NH_2_ and 2-(4-nitrophenyl) benzoxazinone-terminated t-PCL in toluene are prepared. Additionally, the NBD-labeled tracer is added to the t-PEG-NH_2_ (0.67 wt % for 10 kDa t-PEG-NBD and 10 kDa t-PCL-NBD; 1.33 wt % for 20 kDa t-PEG-NBD; 0.33 wt % for 5 kDa t-PEG-NBD), so that the final dye concentration is kept constant for all different tracers. The two solutions are then mixed and left for 72 h at room temperature to allow complete gelation. Note that the tracers did not react and are not covalently attached to the network. For FRS, APCNs were prepared with *Φ* = 0.18–0.30, and for FRAP with *Φ* = 0.03–0.30, by adjusting the solvent fraction.

### Forced Rayleigh Scattering

The APCNs with incorporated dye-labeled tracers are prepared inside FRS sample holders, consisting of two quartz disks with a diameter of 10 mm separated by a 0.5 mm Teflon spacer in a brass sample holder. To prevent solvent evaporation during gelation or measurements, the quartz disks and the Teflon spacer are sealed using commercially available nail polish. After complete gelation, the sample holders are equilibrated at the desired experimental temperature for at least 30 min.

FRS measurements are performed using a setup and methodology adapted from previous designs.^58^ The light source is a Spectra-Physics Cyan 100 mW laser at 488 nm in single-longitudinal mode operation. The beam is split into two beams, focused, and recombined at a scattering angle *θ* on the sample, thereby creating a holographic grating with the characteristic spacing *d*:

1 $$d=\frac{\lambda }{2\cdot \sin\left(\frac{\theta }{2}\right)}$$

Due to constructive interference, a short bleaching beam at maximum intensity with a duration of 100–1000 ms creates an amplitude grating of dye concentration by irreversibly photobleaching the fluorescent dye. This time period is at least an order of magnitude shorter than the diffusion time scales, so the effect of diffusion during writing is negligible. Tracer diffusion can be monitored by a single attenuated reading beam whose diffraction continuously decreases over time due to the decaying intensity profile. A stretched exponential function is then used to fit this decaying intensity:

2 $$I\left(t\right)=A+B\cdot {\exp\left(-{\left(\frac{t}{\tau }\right)}^{\beta }\right)}^{2}$$

with the incoherent scattering background *A*, the amplitude *B*, the stretch exponent *β*, and the characteristic relaxation time *τ*. Using the gamma function *Γ*, the average relaxation time ⟨*τ*⟩ is calculated as

3 $$\left\langle \tau \right\rangle =\frac{\tau }{\beta }\cdot \Gamma \left(\frac{1}{\beta }\right)$$

Each measurement is repeated at least three times. Representative decay profiles and fits are shown in Figure S2.

### Fluorescence Recovery after Photobleaching

The APCNs with incorporated dye-labeled tracers are loaded into a 4 mm thick Teflon spacer flanked by two microscopy quartz glass slides on both sides, sealed with nail polish.

FRAP measurements are performed on a Leica TCS-SP8 microscope with a 10× dry objective of NA 0.3 at 5× zoom. The excitation wavelength is 488 nm (Argon Laser) at 0.05–0.5% of its full intensity. Bleaching is achieved with 100% intensity of the 488 nm laser with a bleaching time of 10 ms for the toluene samples and 1 s for the aqueous samples. The detection wavelength is 498–650 nm. The image resolution is 128 × 128 pixels, resulting in 232.5 × 232.5 μm^2^ images. Scans are performed bidirectionally with a line scanning speed of 1400 Hz. Before bleaching, five images are recorded, averaged, and subtracted from the images recorded after bleaching. After bleaching, two image series are recorded: the first consisting of 500 images and the second of 200 images, the first with a time between images of 0.051 s, and the second with a time between images of 1 s. Analysis of the data is done with a multicomponent diffusion model.^59^ Representative pre- and postbleach images are shown in Figure S3.

### Viscometry

Overlap concentrations of 5 kDa t-PEG and 20 kDa t-PEG in toluene are determined by capillary viscometry using the same convention as for linear polymers:^60^

4 $$\left[\eta \right]=\underset{c\to 0}{\lim}\frac{\eta_{sp}}{c}=\frac{1}{c^{*}}$$

where *η*_sp_ = (*t* – *t*_s_)/*t*_s_ is the specific viscosity, [*η*] is the intrinsic viscosity of the solutions, *t* is the time that a defined volume (0.9 mL) of the solution needs to flow through the capillary, and *t*_s_ is the corresponding time for the pure solvent. The evaluation is carried out according to Schulz and Blaschke.^61^ In detail, a capillary viscometer of micro-Ubbelohde type I is used after sequential cleaning with hydrochloric acid, sodium hydroxide, water, and acetone. Stock solutions of the respective polymers are prepared and diluted to 15, 10, 7, 3, and 1 g·L^–1^. Measurements are performed at 20 °C. The capillary is rinsed with the respective solution and kept at a constant temperature for at least 15 min before the measurements. For each concentration, at least ten data points are collected. The plots according to Schulz and Blaschke are shown in Figure S4.

## Results and Discussion

Let us consider polymer gels at preparation conditions characterized by the correlation length *ξ* inside the gel and tube diameter *a* due to the entanglements at the given polymer volume fraction *Φ*. The dynamics of unentangled polymeric molecules with a size *R* between the correlation length and the tube diameter, *ξ* < *R* < *a*, is then described by the Rouse model^62,63^ for polymers in semidilute solutions. The model predicts the following scaling of the translational diffusion coefficient *D* on molecular mass *M* and polymer volume fraction *Φ*

$$D∼{M^{-1}\Phi }^{-\left(1-\nu \right)/\left(3\nu -1\right)}$$ 5

where *ν* is the Flory exponent. In good solvents (*ν* = 0.588), this corresponds to *D* ∼ Φ^–0.54^, whereas in the concentrated regime (*ν* = 0.5) one expects *D* ∼ *Φ* ^–1^.

For star polymers with a size larger than the tube diameter, *R* > *a*, entanglements are present. Let *M*_e_(1) denote the molar mass of an entangled polymer strand in the melt. In contrast to entangled linear chains with diffusion coefficients scaling as power laws *D* ∼ *M*_e_(1)*M* ^–2^ *Φ* ^–(2−*ν*)/(3*ν*–1)^ and *D* ∼ *M*_e_(1)*M* ^–2^ *Φ* ^–(7/3)^ for the semidilute good solvent and the concentrated regime, respectively, the dynamics of star polymer is exponentially slowed down as a function of molar mass. Star polymers relax primarly by arm retraction, a process involving exponentially rare conformations when the arms withdraw along their confining tubes. Moreover, each retraction allows the star polymers to diffuse only a distance comparable to the tube diameter.^63^ Consequently, the diffusion of entangled star polymers is described by the following expression:

$$D∼M_{e}{\left(1\right)}^{3/2}\cdot M_{a}^{-5/2}\cdot \Phi^{-\left(\frac{5}{2}-\nu \right)/\left(3\nu -1\right)}\cdot \exp\left(-\frac{\gamma^{\prime }}{2}\cdot \frac{M_{a}}{M_{e}\left(1\right)}\cdot \Phi^{1/\left(3\nu -1\right)}\right)$$ 6

Here, *M*_a_ is the molar mass of a star arm and *γ*′ is a numerical coefficient (not far from 3/4 for our sample parameters) describing the effective arm retraction potential.^63^ Thus, in this regime diffusion slows exponentially with increasing *M*_a_ and *Φ*. While in good solvents the blob acts as the effective monomer, in theta solvents the analysis is based on pairwise contacts between chains. Consequently, for theta solvents, the corresponding prediction reads as

7 $$D∼M_{e}{\left(1\right)}^{3/2}\cdot M_{a}^{-5/2}\cdot \Phi^{-3}\cdot \exp\left(-\frac{\gamma^{\prime }}{2}\cdot \frac{M_{a}}{M_{e}\left(1\right)}\cdot \Phi^{4/3}\right)$$

Since the network structure has a decisive influence on the diffusion of solutes, both small and macromolecular, the use of a model APCN with a well-defined structure and a low proportion of inhomogeneities is advantageous for understanding the interplay between structural changes in the network upon solvent exchange.

### Fickian Diffusion of Hydrophilic and Hydrophobic Tracers in APCN in Cosolvent

Forced Rayleigh Scattering (FRS) is a powerful method for examining tracer diffusion in polymer networks across various length scales. By varying the spacing *d* of the holographic grating, specific diffusion regimes can be identified based on the scaling of ⟨τ⟩ ∼ *d* ^2*μ*^. In particular, *μ* = 1 holds for normal Fickian diffusion, whereas *μ* > 1 is consistent with apparent subdiffusion and *μ* < 1 with superdiffusion.

We use FRS to study the diffusion of NBD-labeled 20 kDa tetra-PEG in our PEG–PCL APCNs in the cosolvent toluene at preparation conditions with varying polymer volume fraction of the gel. Figure 2 schematically shows the general sample preparation in toluene. Additionally, we investigate the diffusion of 10 kDa t-PEG-NBD and 10 kDa t-PCL-NBD at a fixed polymer volume fraction of *Φ* = 0.24. Since there are no associative interactions to be expected between the dye-labeled tracers and the surrounding polymer network, we need to ensure that the tracers are slowed down enough to be well observed by using a relatively high polymer volume fraction. The diffusion can be studied on length scales from 2.8–41.9 μm. At smaller length scales, diffusion would still be too fast to be accurately monitored. Fitting of the obtained relaxation times ⟨*τ*⟩ vs *d* ^2^ yields a slope of 1 within the margin of error as shown in Figure 3, indicating purely Fickian diffusion on the examined length scales, thereby matching expectations. A pronounced discontinuity appears between the samples at *Φ* = 0.27 and 0.285, suggesting that additional mechanisms begin to influence the relaxation dynamics at higher concentrations. The precise origin of this transition, however, remains unresolved so far. However, confirming the absence of anomalous behavior in the investigated systems is the key outcome of the FRS experiments and provides the basis for the subsequent discussion of the data and the underlying diffusion processes in terms of scaling relationships.

**Figure 2:**
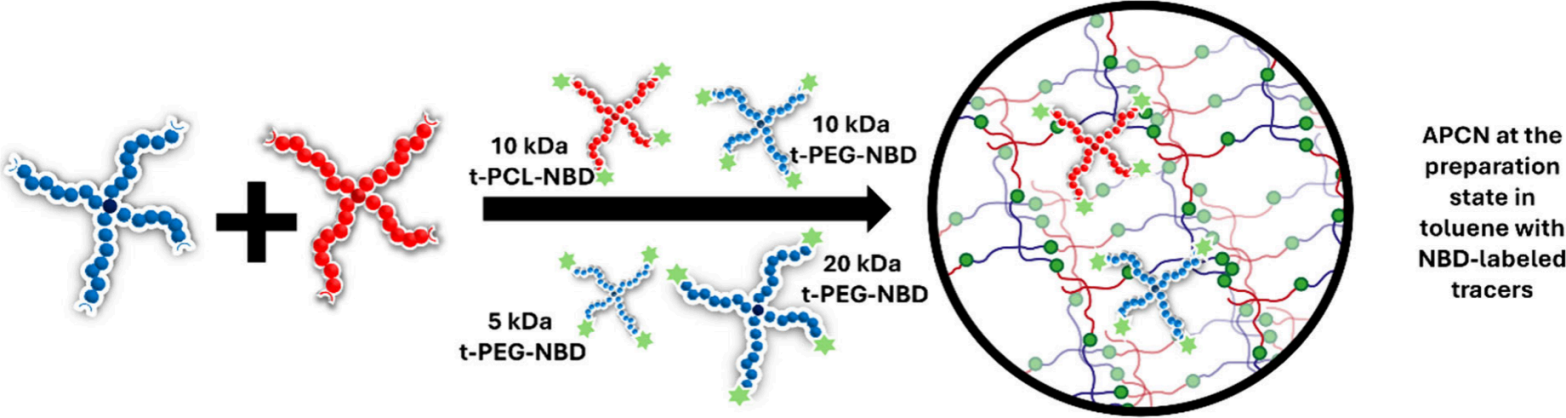
Schematic presentation of sample preparation in toluene. Amino-terminated t-PEG and 2-(4-nitrophenyl) benzoxazinone-terminated t-PCL are dissolved in toluene containing one of the fluorescent tracer star polymers, and the network forms by heterocomplementary coupling, while the dye-labeled tracers remain unreacted inside the gel.

**Figure 3:**
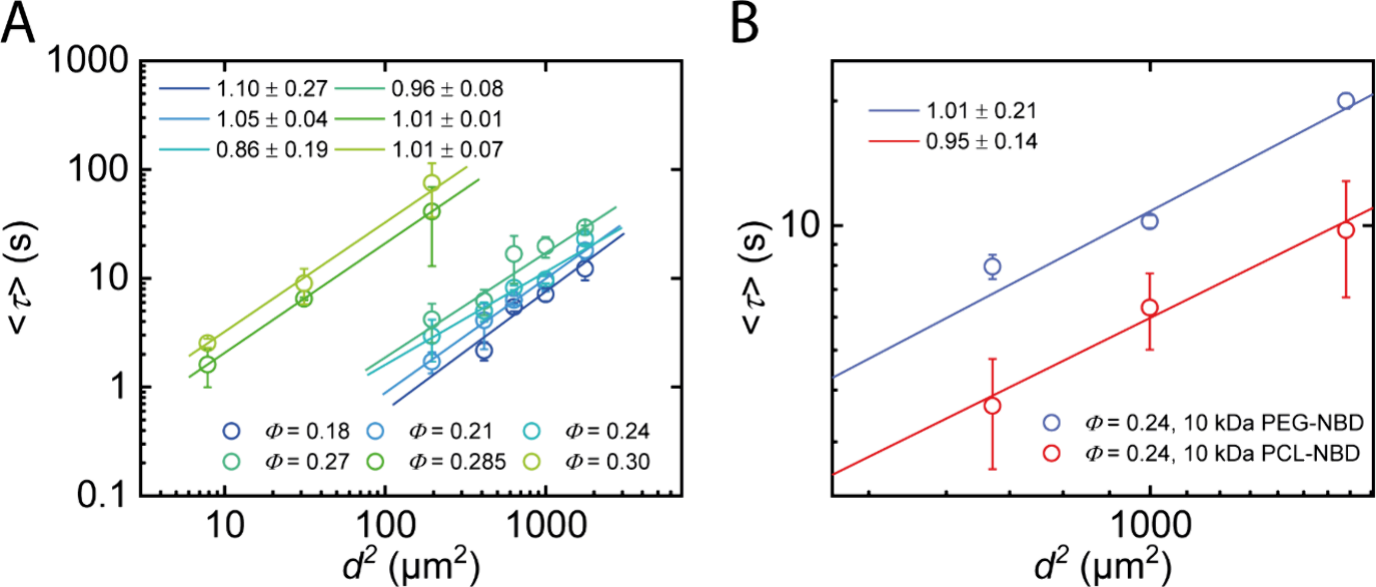
FRS experiments reveal Fickian diffusion of (A) 20 kDa t-PEG-NBD in APCNs at various polymer volume fractions and (B) 10 kDa t-PEG-NBD and 10 kDa t-PCL-NBD in an APCN at *Φ* = 0.24. All measurements are performed at 10 °C to slow down the diffusion of the tracers.

### Polymer Volume Fraction Dependence of Hydrophilic and Hydrophobic Tracers

The diffusion coefficients can be obtained from the relaxation time ⟨*τ*⟩ as determined by eq 3 and the spacing *d* as determined by eq 1 using the following relation:

8 $$D=\frac{d^{2}}{4\pi^{2}\left\langle \tau \right\rangle }$$

With an increasing polymer volume fraction, the decay of the holographic grating takes longer, indicating a slower diffusion of the 20 kDa t-PEG-NBD in a denser network. The diffusion coefficient of hydrophobic 10 kDa t-PCL is larger than that of hydrophilic 10 kDa t-PEG at the same molecular weight in a network at *Φ* = 0.24. This is somewhat surprising as Bunk et al. determined overlap concentrations of these star polymers in the same solvent that point toward larger PCL stars in the dilute limit. However, we have to note that our experiments were conducted at 10 °C and that the size of the t-PEG star polymers in toluene significantly shrinks for decreasing temperature, whereas the size of the t-PCL seems to be less temperature-sensitive, as the data for the overlap concentrations of Bunk et al. shows.^46^ Thus, the PEG chains might even start to become sticky at the low temperatures of the FRS experiments, and we have to consider these data with some care.

To extend the investigated polymer volume fraction range to *Φ* = 0.03–0.3, we use FRAP at room temperature with 10 kDa t-PEG-NBD as tracer. Since the length scale of the FRAP experiments is in the range of the investigated length scales from FRS experiments, we can assume Fickian diffusion for the FRAP experiments. In Figure 4, the obtained diffusion coefficients are plotted against the polymer volume fraction of the APCN. Fitting of the data with a power law gives a scaling exponent of – 0.60 ± 0.04 for low polymer volume fractions, marginally steeper than the expected Rouse model prediction for the semidilute good solvent regime.

**Figure 4:**
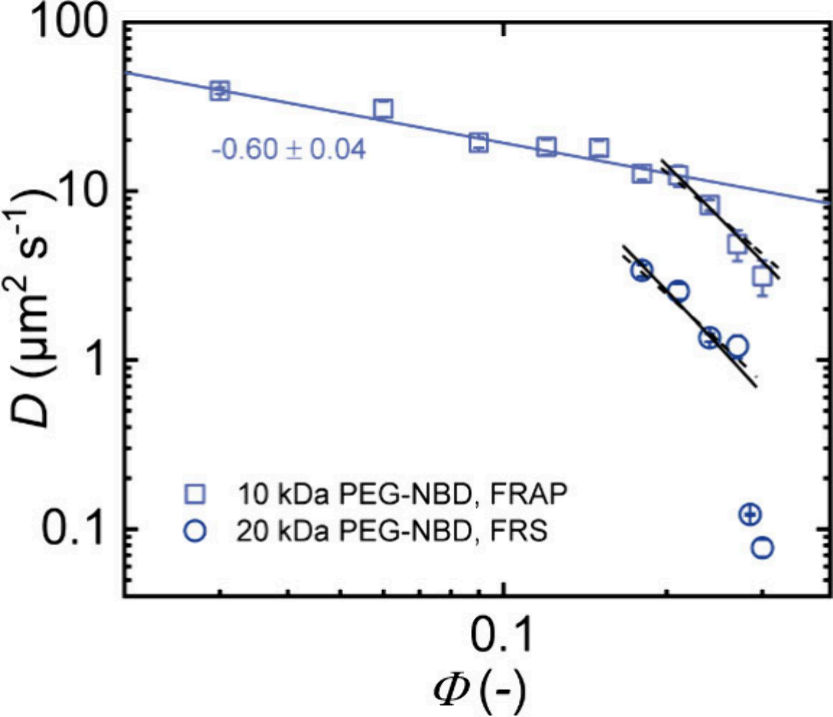
Diffusion coefficients *D* of 10 kDa t-PEG obtained from FRAP and 20 kDa t-PEG from FRS for APCNs in toluene prepared at different polymer volume fractions with a respective power law fit for the unentangled regime and fits according to dynamics of entangled stars for higher polymer volume fractions with eqs 6 and 7 for the good solvents (black, dashed lines) and the concentrated (black, solid lines) regimes, respectively.

At *Φ* ≈ 0.2, we observe a transition to steeper scaling due to the appearance of entanglements. From viscosity measurements, Bunk et al. determined *χ*_η_ = 0.38 for 10 kDa t-PEG in toluene.^46^ With this, we can estimate the transition to the concentrated regime Φ** according to Φ** ≈ *v*/*b* ^3^ ≈ 1 – 2*χ*_η_ ≈ 0.24. Thus, the transition between the good solvent and the concentrated regime is roughly at the same place as the transition between the nonentangled and the entangled regime. For a better comparison, we fit both sets of data with predictions for both the good solvent and the concentrated regime, respectively. Due to the narrow range of concentrations, no clear distinction can be made; however, there is a tendency that the 10 kDa t-PEG data agree better with the prediction for the concentrated regime. For the 20 kDa t-PEG measured with FRS at 10 °C, the data at the largest concentrations drop significantly below the prediction for the entangled star polymers, clearly indicating that additional processes must be active, slowing the diffusion. The little discrepancy between this abrupt transition and the predicted crossover estimated from literature is in the range of the accuracy of our experimental data.

Additionally, we studied the diffusion of NBD-labeled hydrophobic 10 kDa t–PCL inside the network in toluene at different polymer volume fractions. Again, we vary the polymer volume fraction of the network and find a weaker concentration dependence as predicted by the Rouse model followed by a possible transition to the entangled regime around *Φ* ≈ 0.30, see Figure 5. Bunk et al. reported interaction parameters of *χ*_η,PEG_ = 0.38 and *χ*_η,PCL_ = −0.05^46^, whereas we estimated the PEG–PCL interaction parameter according to Hansen to be *χ*_PEG,PCL_ = 0.15, with solubility parameters taken from refs 46 and 65. Thus, for increasing polymer volume fraction, the PCL stars lose contacts with the more favorable solvent on top of a decreasing entropic contribution, which reduces their size and increases their mobility. For PEG, increasing the polymer volume fraction refers to an increase in the effective interaction, which enhances the concentration dependence of diffusion. This contrary solution behavior explains qualitatively the differences in the observed scaling of the diffusion data in the unentangled semidilute good solvent limit. In general, we expect similar deviations from the ideal Rouse behavior (derived for a homopolymer solution) for the diffusion of any polymeric solute in a conetwork or in a network made by a different polymer. The reason is that for increasing concentrations, contacts with the solvent are replaced by contacts with the surrounding polymer network, which modifies the effective excluded volume parameter and thus the size of the diffusing molecules. Nevertheless, given the limited number of data points and relatively narrow concentration range, the observed trends should be interpreted with some caution.

**Figure 5:**
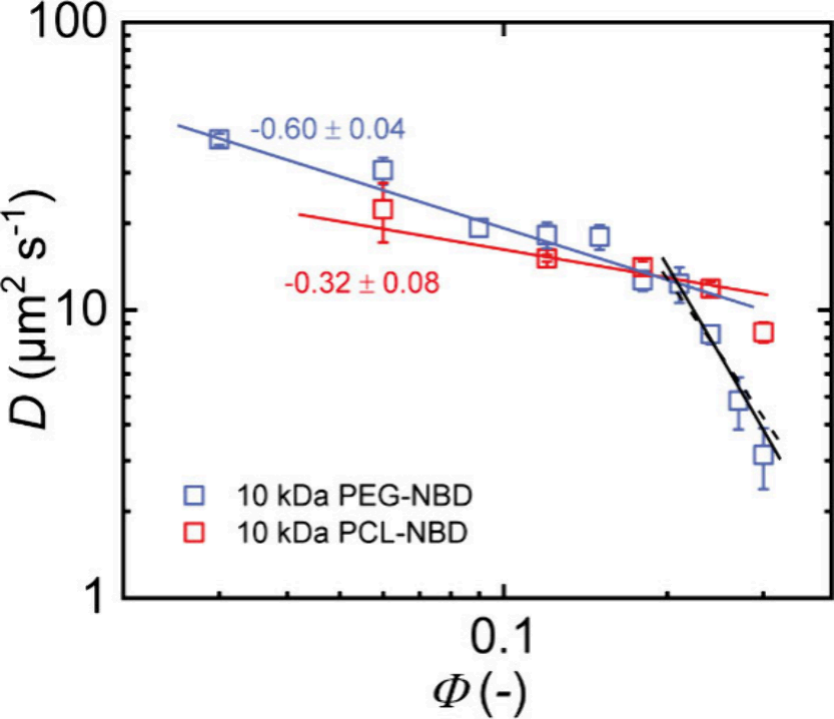
Diffusion coefficients *D* for the diffusion of 10 kDa t-PCL from FRAP experiments for APCNs in toluene at varied polymer volume fractions. Fitting with a power law reveals a weaker dependence than predicted by Rouse model. For better clarity and comparability, the diffusion data of 10 kDa t-PEG are also shown.

We have to note that the crossover to entangled dynamics is expected to occur at concentrations somewhat higher than those observed in our experiments. Based on the textbook scaling of the entanglement degree of polymerization with concentration and literature data for *N*_e_,^63^ the transition to entangled dynamics is predicted at around *Φ* ≈ 0.42, which is noticeably higher than the concentrations at which we observe the crossover experimentally. This could be related to the fact that the transition occurs actually from the unentangled to the strangulated regime^66,67^ instead of the entangled regime or that we experience a mixed transition where both the strangulation regime and the entangled regime contribute, since the observed and expected transition concentration differ by only a factor of roughly two. In the strangulation regime, the network spacing is smaller than the entanglement length, and the polymer stars are effectively confined in narrower tubes, leading to diffusion slower than that in the unentangled Rouse regime. Entangled dynamics, in contrast, require a network spacing and polymers larger than the entanglement length.^67^ A clear distinction would be possible, if a broad entangled or strangulated regime (as a function of concentration) would be available, since both strand size and blob size scale differently in semidilute solutions leading to a distinct scaling law for the diffusion of solutes.

Independent of the mechanism, we have to emphasize that it is the network that sets either the tube diameter or the strangulation length. Thus, this parameter is independent of the particular tracer polymer. A tracer will exhibit entangled (or strangulated) dynamics only if its size exceeds the tube diameter (or the strangulation length). This is visible in good approximation in Figure 5, where the crossover to the entangled behavior occurs in a narrow range of polymer volume fractions (thereby defining a narrow range of tube diameters) where the tracers of roughly the same size (as visible in the diffusion coefficients) start to entangle.

### Molecular Weight Dependency of Hydrophilic Tracers

With FRAP, we also study the diffusion of t-PEG tracers at varying molecular weights in networks at the overlap concentration (*Φ* ≈ 0.06), where we can expect to be in the unentangled regime even for higher molecular weights, and at *Φ* ≈ 0.18, which is somewhat below the experimentally determined onset of entanglement effects for 10 kDa t-PEG. Following expectations, we observe a scaling exponent of −0.95 ± 0.12 for the molecular weight scaling of the diffusion coefficient in the network prepared at the overlap concentration, as shown in Figure 6.

**Figure 6:**
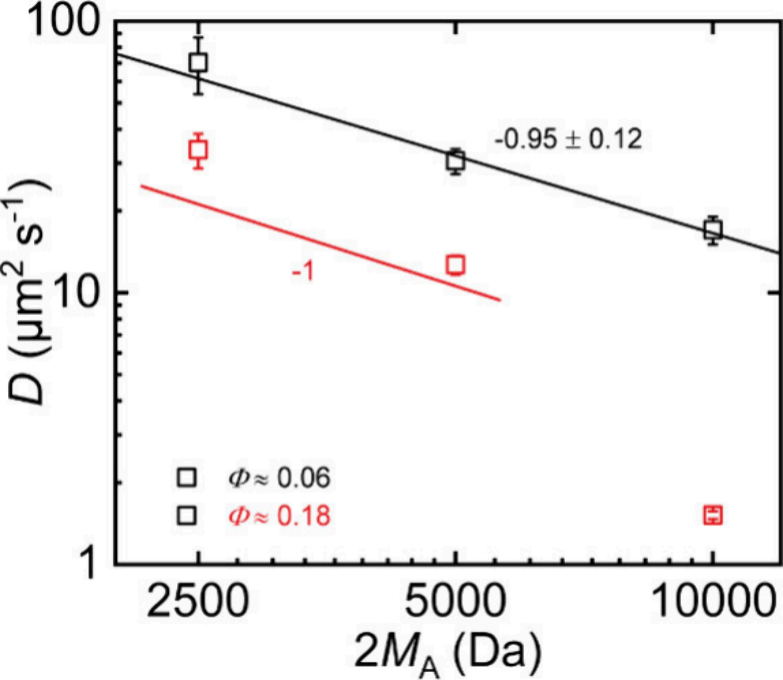
Diffusion coefficients *D* obtained from FRAP experiments with t-PEG tracers with varying molecular weight. The black line is a power law fit to the data at *Φ* ≈ 0.06, the red line indicates the scaling of unentangled star polymers in the networks with *Φ* ≈ 0.18.

For the networks prepared at *Φ* ≈ 0.18, the data cannot be just fitted by a simple power law. While the overall trend reflects slower diffusion for larger tracers, the curvature of the plot supports our observation of an onset of entanglement effects (or strangulation dynamics) around *Φ* ≈ 0.24 for the 10 kDa stars. The entanglement threshold is approximately 1.7 times lower for stars with twice the molar mass, such that these start to develop entangled dynamics at *Φ* ≈ 0.18. Since the expected shift for the onset of the strangulated regime is not largely different, we cannot conclude from these data in favor of one or the other mechanism.

## Diffusion of Hydrophilic Tracers in APCN Swollen in a Selective Solvent

The investigation of t-PEG diffusion in the APCN swollen in selective solvent water is of great interest due to the potential applications of this medium. In water, the PCL collapses due to its hydrophobicity, forming clusters, as it has been shown by Löser et al.^19^ For possible applications, we expect two different insights to be of particular interest: first, how the diffusion changes upon a change in the swelling degree of the APCN from a minimal swelling degree to the equilibrium swelling degree. And second, how the diffusion changes when a solvent exchange from a cosolvent to a selective solvent happens for APCNs prepared at different polymer volume fractions.

Again, the solute studied is 10 kDa t-PEG-NBD, allowing for a more detailed comparison of diffusion between the APCN swollen in the cosolvent toluene and the selective solvent water. First, we investigate the diffusion in networks prepared at five different initial polymer volume fractions in toluene (*Φ*_prep_ = 0.06–0.30). The networks were swollen to equilibrium in toluene to eliminate any residual sol phase after preparation and then dried and swollen in water to equilibrium (variant 1, see Figure 7). The resulting equilibrium degrees of swelling, *Φ* = 2.95 ± 0.03,^68^ due to the collapse of the PCL phase, refer to a polymer volume fraction of the swollen PEG phase of approximately *Φ*_PEG_ ≈ 0.18, which is below the onset of entanglement effects at around *Φ* ≈ 0.24. Therefore, we expect that dynamics will not couple to entanglements. In the unentangled regime, the correlation length *ξ* is controlling diffusion. Since the polymer volume fractions at swelling equilibrium are roughly equal across all samples,^19,68^ we expect a diffusion that is mainly independent of the preparation conditions in agreement with the data in Figure 8. An average diffusion coefficient of *D*_water_ = 43.2 ± 1.6 μm^2^/s is obtained.

**Figure 7:**
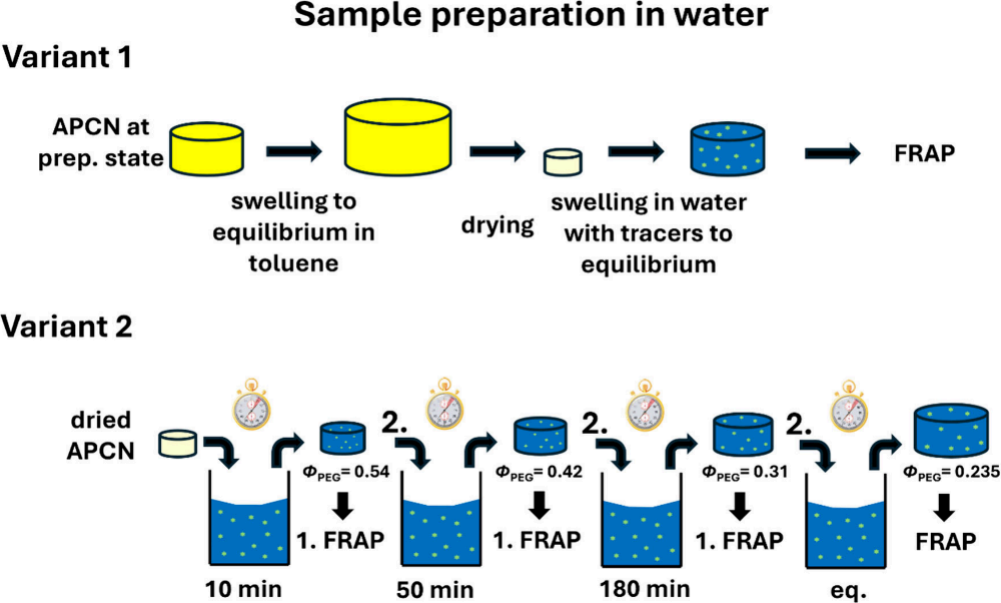
Schematic presentation of the different sample preparation variants used in water. In all cases, the APCN is first prepared in toluene without any dye-labeled tracers, swollen to equilibrium in toluene and then dried. For variant 1, the dried APCNs are placed in an aqueous solution containing the t-PEG-NBD and allowed to swell to equilibrium before FRAP measurements. In variant 2, the dried APCN is placed in an aqueous solution containing t-PEG-NBD, removed after 10 min for FRAP analysis, then replaced in the solution for 50 min and analyzed again, followed by another 180 min incubation and FRAP measurement, before finally swelling to equilibrium and performing a last FRAP analysis.

**Figure 8:**
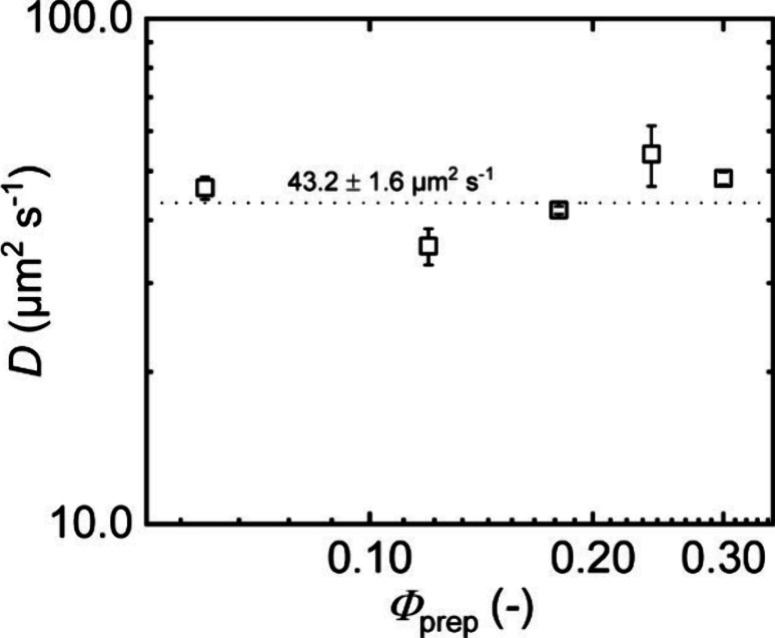
Diffusion coefficients *D* obtained from the diffusion of 10 kDa t-PEG in APCNs prepared at different polymer volume fractions *Φ*_prep_ in toluene, then dried and reswollen in water to an equilibrium swelling degree of roughly 3 across all samples.

Further support for this observation stems from preceding work on networks that are equivalent to ours. The water-swollen APCNs have been analyzed previously with SAXS and bond-fluctuation model simulations by Löser et al.,^19^ who found nearly constant values for both the average distance and the radii of the microphase-separated PCL clusters across a wide range of preparation concentrations. As both the degree of swelling and the size of PCL clusters are independent of the initial polymer volume fraction, we can assume that the local t-PEG concentration between the PCL clusters is also independent of the initial polymer volume fraction. Therefore, the tracers experience the same diffusion-hindering factors in all APCNs at swelling equilibrium, leading to a diffusion coefficient that is independent of *Φ*_prep_.

To investigate the dependence of the diffusion coefficient on the swelling degree and thus on measurement conditions different from preparation conditions, we prepared an APCN at a concentration of 350 g/L in toluene. After complete gelation, the gel is swollen to equilibrium in toluene to eliminate any residual sol phase. In the next step, the gel is thoroughly dried under vacuum at room temperature and then reimmersed in water to reach the desired swelling degrees (variant 2, see Figure 7). For this, the sample is removed from the aqueous tracer solution for FRAP measurements at specific time points and returned to the solution afterward to allow for further swelling. Again, the tracer studied is 10 kDa t-PEG-NBD. Since time-controlled swelling can, in principle, lead to spatial gradients in the polymer volume fraction, one might expect such effects. However, during the FRAP measurements, no spatial fluorescence intensity gradients were observed across the samples, indicating a homogeneous tracer distribution and the absence of detectable swelling gradients at the time of the analysis. The diffusion data is plotted as a function of the PEG volume fraction in the swollen part of the gel, since the collapsed PCL domains are not accessible for the PEG tracers, see Figure 9. Since the preparation conditions and the measurement concentrations refer to the entangled limit, we compare the data with two qualitatively different model predictions. First, we take eq 7 for the concentrated regime of entangled star polymers assuming that upon drying, additional temporary entanglements form that are relevant for diffusion. An alternate estimate is obtained by assuming that the entanglement network is entirely fixed at preparation conditions and that the tube diameter deforms affinely with the sample volume (exactly the same reasoning would apply also for the strangulation regime). In this latter case, we obtain

9 $$D∼D_{prep}\Phi^{-2}\exp\left(-\frac{\gamma^{\prime }}{2}\cdot \frac{M_{a}}{M_{e}\left(\Phi_{0}\right)}\cdot {\frac{\Phi }{\Phi \left(0\right)}}^{2/3}\right)$$

**Figure 9:**
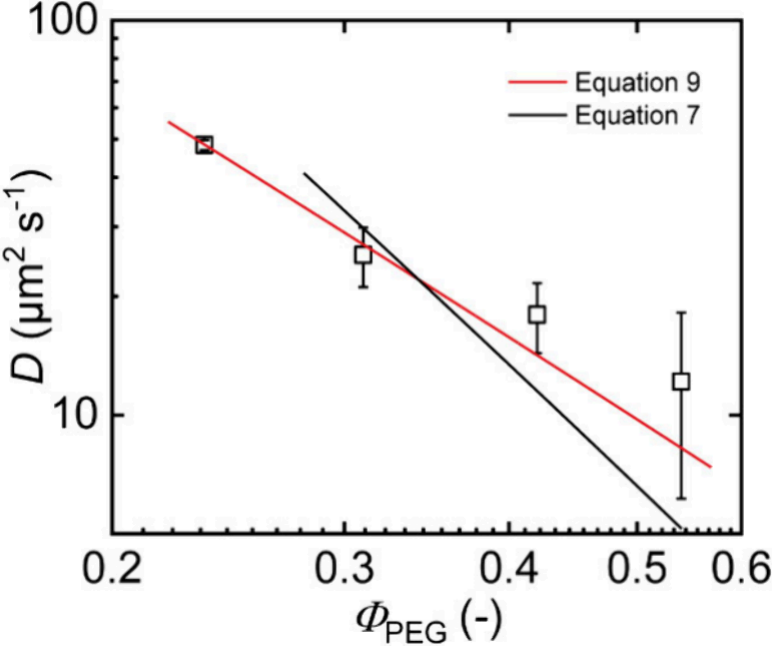
Diffusion coefficients *D* obtained from the diffusion of 10 kDa t-PEG in APCNs prepared at *Φ*_prep_ = 0.30 in toluene, then dried and reswollen in water, and studied as a function of the PEG volume fraction in the swollen PEG phase of the gels. The lines indicate a fit of the data to eq 7 and eq 9 that considers an affine swelling of the entanglement network.

We expect, that *N*_a_/*N*_e_ is close to unity and use *γ’* = 3/4 for a low number of entanglements per arm.^63^ The comparison with the experimental data indicates that an affine deformation of the constraints seems to provide a better approximation of the data even for drying the samples from the preparation conditions, which indicates that our network could be in the strangulation regime. This agrees with diffusion experiments by Rotstein et al. that also suggest an affine deformation of the constraints during network swelling.^69^ However, we note that the measured diffusion coefficients clearly exceed the corresponding results under preparation conditions. Clearly, the PEG phase is not homogeneous around the PCL domains with largely enhanced PEG concentrations next to the interface that decays rapidly toward those parts of the PEG phase that are further away from the interface. Since transport is dominated by the regions with the fastest diffusion, quantitatively, the observed diffusion coefficients agree better with the data in the unentangled limit; however, the concentration scaling is clearly stronger due to additional constraints like entanglements or confining network strands like in the strangulation regime. Altogether, a definite conclusion cannot be drawn as to whether our data refer to the strangulation regime or result from a superposition of entanglement effects and sample heterogeneity. Therefore, future measurements covering a broader range of molar masses, concentrations, and different solvents are required to clarify the situation.

### Diffusion Data Normalized to Tracer Overlap Concentration

In a previous work, Cherdhirankorn et al. demonstrated that the diffusion data of labeled polystyrene tracers of various molecular weights in unlabeled polystyrene solutions at different polymer concentrations superimpose on a single curve upon normalizing the diffusion coefficient to that of the tracer in pure solvent and adjusting the matrix concentration to the overlap concentration of the tracer.^70^ In line with that, in Figure 10, we plot the diffusion coefficients obtained from FRAP and FRS measurements of t-PEG tracers versus the polymer volume fraction of either PEG in the PEG phase (swelling in water) or the full network (swelling in toluene). All of these volume fractions were normalized with respect to the tracer overlap concentration *Φ*_p_*. Tracer overlap concentrations were either determined in this work (*Φ*_p_*­(5 kDa t-PEG, toluene) = 146 ± 18 g/L and *Φ*_p_*­(20 kDa t-PEG, toluene) = 40.3 ± 0.2 g/L; see Figure S3) or used as reported in the literature (*Φ*_p_*­(10 kDa t-PEG, toluene) = 76.2 ± 0.5 g/L;^46^ *Φ*_p_*­(10 kDa t-PEG, water) = 60 ± 2 g/L).^68^ Also, we normalized the diffusion coefficients to the viscosity of the respective solvents, *η*_s_ at the particular temperature. Almost all diffusion data of t-PEG inside the APCN swollen in toluene, independent of the method or the tracer molecular weight, collapse onto one curve. The expected exception is the 5 kDa data, since it cannot couple to the larger correlation length of the network at the overlap concentration of the larger stars that establish the network. Such a collapse is only possible if all network concentrations remain within one concentration regime (either semidilute good or concentrated). In our case, the data at the largest concentrations are only weakly entering this regime only marginally affecting the quality of the collapse. Moreover, the FRS data measured at lower temperature does not fully collapse with the FRAP data. Here, the temperature dependence of the interaction parameter seems to cause a weak shift of the data. The data of the samples selectively swollen in water show a clearly different behavior for reasons explained in the preceding section. We conclude that not only the measurement concentration, but also the preparation concentration and the homogeneity of the samples play a vital role in the transport properties. Moreover, we excluded the t-PCL from this plot due to a modified scaling of the data in the nonentangled regime that certainly does not allow for a data collapse (for comparison, see Figure S5).

**Figure 10:**
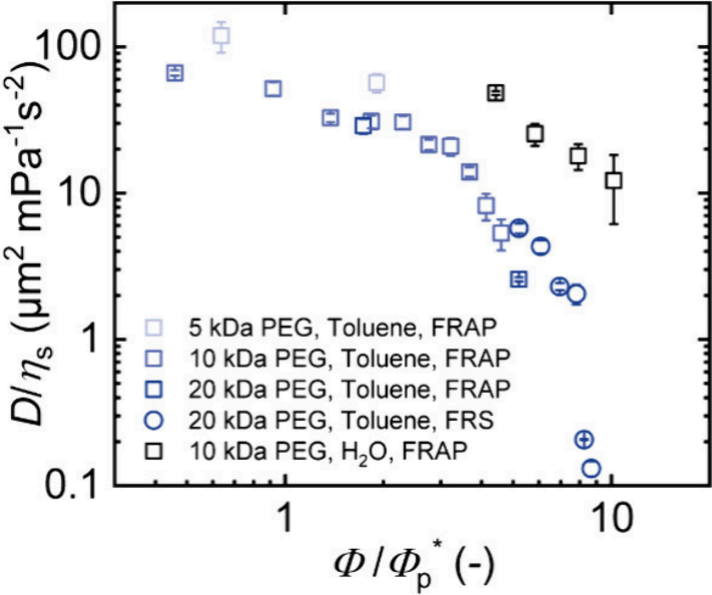
Diffusion coefficients *D* normalized to the solvent viscosity *η*_s_ plotted against the polymer volume fraction *Φ* normalized to the tracer overlap concentration *Φ*_p_*.

Altogether, our results indicate that preparation conditions, the conditions at measurement including the homogeneity of the samples and a possible swelling or deswelling of the entanglement network or the network meshes as in the strangulation regime can play a vital role for the diffusion behavior.

## Conclusions

In this study, PEG–PCL model amphiphilic polymer conetworks are prepared using heterocomplementary cross-linking and investigated regarding the diffusion of hydrophilic t-PEG and hydrophobic t-PCL tracers inside the APCNs swollen in either the cosolvent toluene or the selective solvent water using FRS and FRAP. The APCN swollen in toluene allows for the diffusion of hydrophilic and hydrophobic tracers. FRS experiments confirm the absence of anomalous diffusion; as expected, all investigated tracers exhibit Fickian diffusion. FRAP enables the study of faster tracer diffusion, allowing the investigation of diffusion in APCNs with lower polymer volume fractions, providing valuable insights into the underlying diffusion mechanisms. For 10 kDa t-PEG, we observe a transition from the unentangled regime with Rouse dynamics to an entangled or strangulation regime; however, a clear distinction is not possible with the data at hand. For t-PCL, we observe Rouse diffusion with a possible transition to a slower regime at the largest polymer volume fractions. The observed entanglement polymer volume fraction for 10 kDa t-PEG is roughly a factor of 2 lower than expected from the entanglement molar mass in melts, which indicates that the data may lie in (or are affected by a crossover to) the strangulation regime proposed by Antonietti and Sillescu.^66^ Moreover, diffusion measurements with t-PEG tracers of varying molecular weight in APCNs at the overlap concentration followed the scaling predicted by the Rouse model. The same experiment in an APCN prepared at *Φ* ≈ 0.18 shows a transition from Rouse scaling to a steeper scaling, indicating either the onset of entanglements or the strangulation regime. The diffusion coefficient of 10 kDa t-PEG in APCNs swollen to equilibrium in the selective solvent water is found to be independent of the initial polymer volume fraction used during network formation in toluene. Even if this finding seems counterintuitive, this is consistent with an identical microstructure^19^ and equilibrium swelling degree across all preparation polymer volume fractions.^68^ For the scaling of the tracer diffusion inside an APCN in water at different swelling degrees and thus different polymer volume fractions, it agrees best with the assumption of an affine swelling of the network strands or the tube diameter. However, a quantitative comparison indicates a clear impact of sample heterogeneity. Therefore, additional investigations are needed to improve our understanding of diffusion in APCNs.

With this, we demonstrate the suitability of APCNs for the diffusion of hydrophilic and hydrophobic polymers and provide insights into the underlying diffusion mechanisms. Furthermore, these results highlight the conditions for selective diffusion in APCNs. In toluene-swollen samples, t-PEG diffuses faster than t-PCL at low polymer volume fractions, whereas at higher volume fractions, t-PCL becomes relatively faster, reflecting the influence of solvent quality in this ternary system. In water-swollen networks, t-PCL is insoluble, allowing only for the diffusion of hydrophilic t-PEG. These findings demonstrate that the relative mobility of hydrophilic and hydrophobic polymeric tracers can be tuned by the solvent quality and network composition. This marks a further step toward designing controlled polymer-diffusion-based drug delivery systems for biomedical applications.

Overall, these results demonstrate that selective diffusion in APCNs can be tuned through solvent conditions and network composition, allowing for control over the relative mobility of hydrophilic and hydrophobic species. Such tunability provides guidance for designing polymer networks with controlled, polarity-, and composition-dependent transport, which is relevant for applications in drug delivery, biomaterials, and other systems where selective or targeted transport is desired.

## Supplementary Material



## Notes

Additional figures, including FRS decay curves, representative FRAP images, and viscometry data (PDF)
