# Supplementary material for: Tracer Diffusivity in Amphiphilic Polymer Model Co-Networks
Source: Macromolecules. 2026 Jan 16;59(3):1282–92. doi: 10.1021/acs.macromol.5c02458 (PMC12895526; doi:10.1021/acs.macromol.5c02458)
Supplement: Supplementary file 1 [file ma5c02458_si_001.pdf]

Supporting Information

Tracer Diffusivity in Amphiphilic Polymer Model Co-Networks

Sebastian Seitel, Lynn K. R. J. Zank, Stephanie Ihmann, Frank Böhme, Michael Lang, Bradley D. Olsen, Sebastian Seiffert**

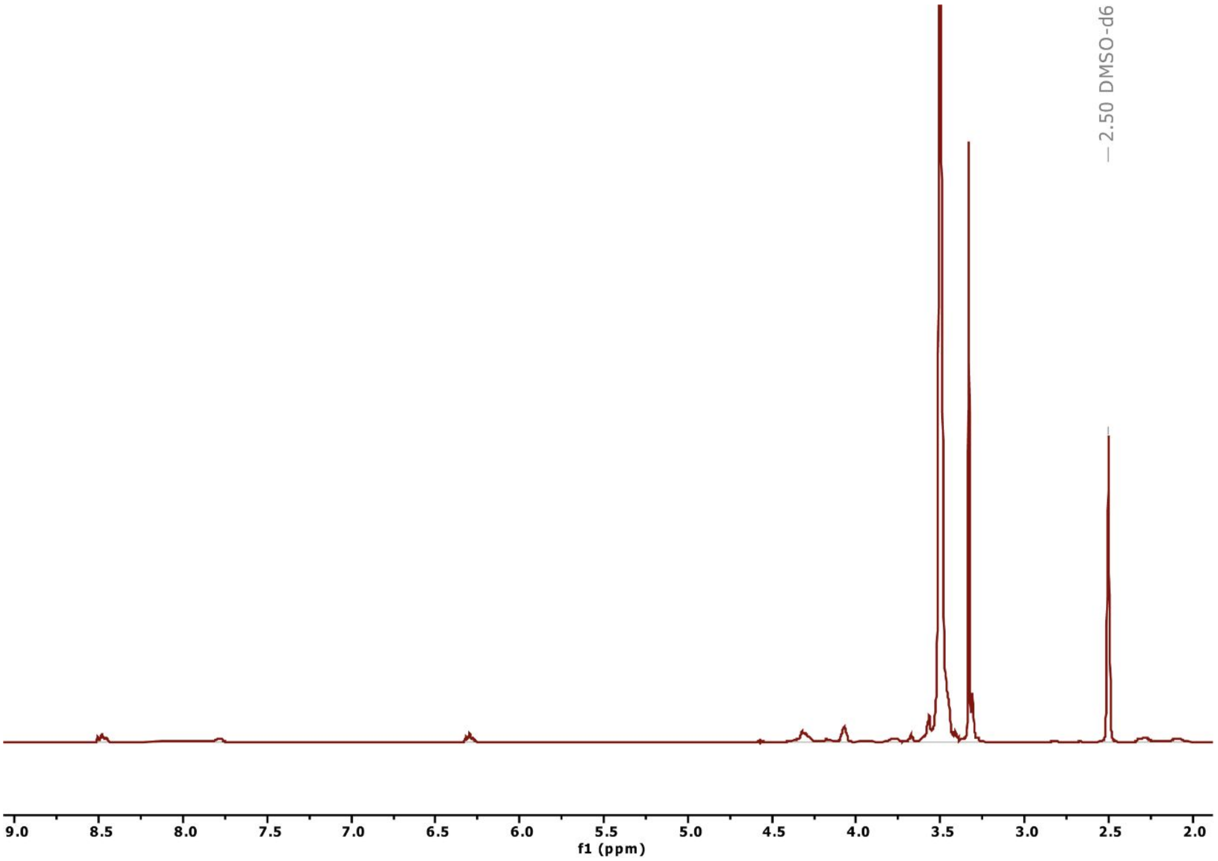

Figure S1. ^{1}H NMR spectrum of 5 kDa t-PEG-NBD in DMSO-d_6.

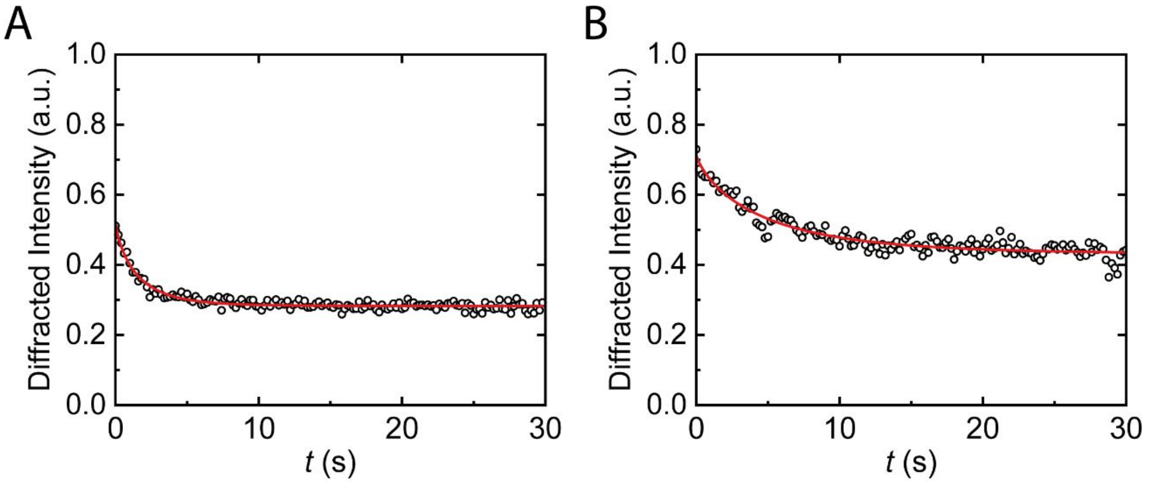

Figure S2. Representative decays of the diffracted scattering intensity over time as probed by FRS for the diffusion of 10 kDa t-PEG-NBD in a model PEG-PCL APCN in toluene prepared at $\Phi = 0.24$. The black open circles are experimental data, and the red lines are fits to a stretched monoexponential function. (A) Tracer diffusion measurement at a beam angle of 2 degrees. (B) Tracer diffusion measurement at a beam angle of 1.11 degrees. Both measurements were performed at 10 ˚C.

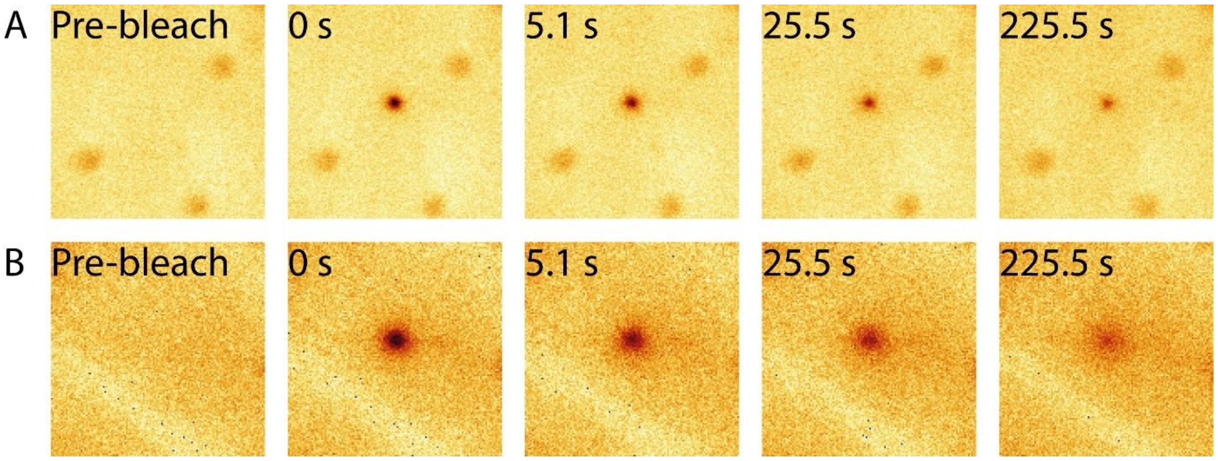

Figure S3. Representative FRAP measurements of the diffusion of 10 kDa t-PEG-NBD in A) a PEG-PCL APCN swollen in toluene prepared at Φ = 0.18, and B) the same APCN, but thoroughly dried and then reswollen in water to swelling equilibrium.

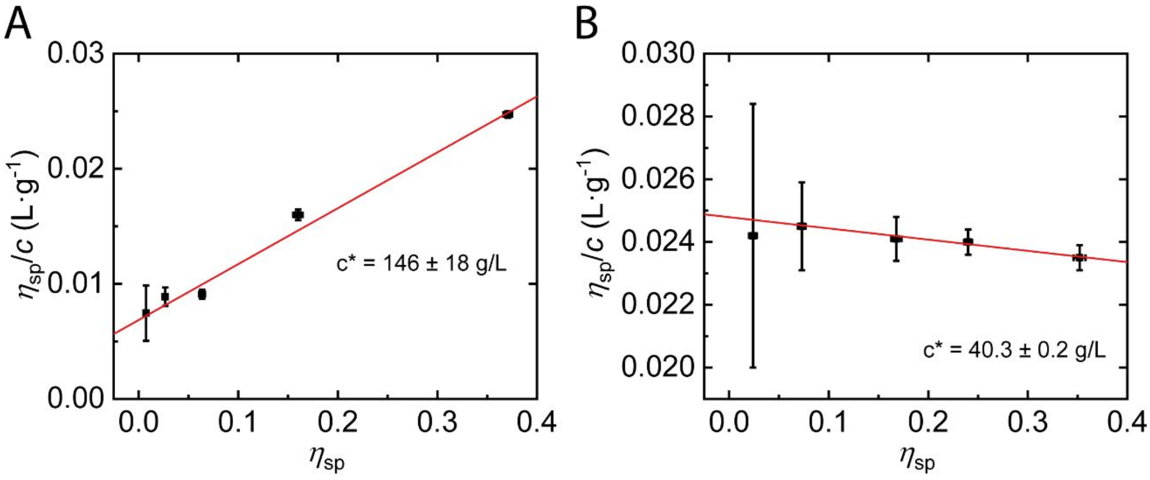

Figure S4. Evaluation of the viscometry measurements according to Schulz and Blaschke to determine the overlap concentration in toluene of A) 5 kDa t-PEG, and B) 20 kDa t-PEG. Both measurements were performed at 20 ˚C.

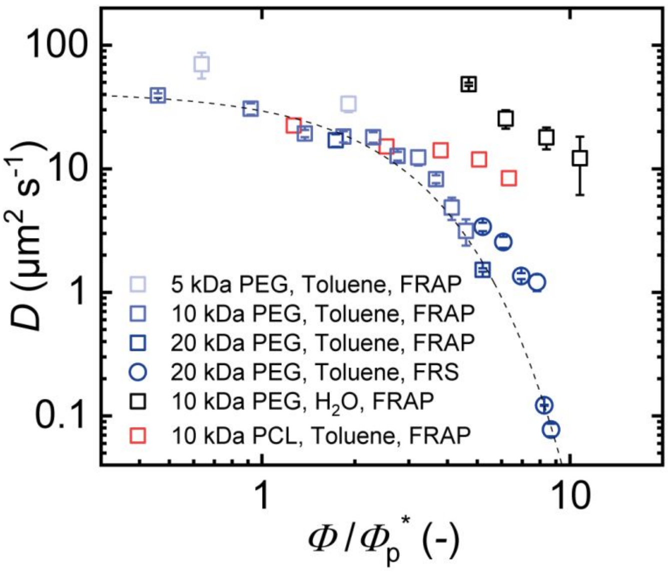

Figure S5. Diffusion coefficients D of t-PEG and t-PCL normalized to the solvent viscosity η_s plotted against the polymer volume fraction Φ normalized to the tracer overlap concentration $\Phi_p{}^*$.

AUTHOR INFORMATION

Corresponding Authors

*Sebastian Seiffert – *Johannes Gutenberg University Mainz, Department of Chemistry, D-55128 Mainz, Germany;* orcid.org/0000-0002-5152-1207; Email: sebastian.seiffert@uni-mainz.de

*Bradley D. Olsen – *Massachusetts Institute of Technology, Department of Chemical Engineering, Cambridge, MA 02139, United States;* orcid.org/0000-0002-7272-7140; Email: bdolsen@mit.edu

Authors

Sebastian Seitel – *Johannes Gutenberg University Mainz, Department of Chemistry, D-55128 Mainz, Germany; Massachusetts Institute of Technology, Department of Chemical Engineering, Cambridge, MA 02139, United States;* orcid.org/0009-0004-6326-0632

Lynn K. R. J. Zank – *Johannes Gutenberg University Mainz, Department of Chemistry, D-55128 Mainz, Germany;* orcid.org/0009-0000-2674-0013

Stephanie Ihmann – *Leibniz-Institut für Polymerforschung, 01069 Dresden, Germany; Organic Chemistry of Polymers, Technical University Dresden, 01062 Dresden, Germany;* orcid.org/0009-0004-7724-6372

Frank Böhme – *Leibniz-Institut für Polymerforschung, 01069 Dresden, Germany;* orcid.org/0000-0001-6128-4658

Michael Lang – *Leibniz-Institut für Polymerforschung, 01069 Dresden, Germany;* orcid.org/0000-0003-3851-6670
